# The Use of Arsenious Acid for Destroying the Nerve in Decayed Teeth

**Published:** 1842-03

**Authors:** W. E. Ide

**Affiliations:** Zanesville, Ohio.


### ARTICLE III.

*The use of Arsenious Acid for destroying the Nerve in Decayed Teeth.* By W. E. Ide, M. D., of Zanesville, Ohio.

A safe and efficient agent for destroying the nerves of decayed and painful teeth, has ever been a desideratum. Among the various things that have at different times been suggested for this purpose, none has been more unqualifiedly lauded by some, and condemned by others, than arsenic. In the "Guide to Sound Teeth," published by Dr. Spooner, of New York, in 1836, public attention is first called to its use; and its qualities are extolled in no measured terms. In a work soon after published by Dr.

Burdell, its use is condemned as cruel, ineffectual and dangerous. In vol. 1, page 227, of the Journal of Dental Science, Dr. S. Brown says—"of all the remedies which have hitherto been generally made known to the profession in this country, arsenic is unquestionably the most prompt, safe, and effectual, when properly applied." The opinions of such men as these, surely no one acquainted with his profession will treat with disrespect: but as "the doctors disagree," it may be difficult for the inexperienced to decide. As the matter now stands, we fear that some may wholly discard a valuable remedy, while others may use it so indiscriminately, as frequently to fail of the beneficial results that might otherwise be obtained. In the hands of the above mentioned gentlemen who have recommended it, it has undoubtedly been so judiciously used, as to be unattended by the objections that have elicited this communication: but others, as we have done, may use it in a manner to make a caution not altogether unnecessary.

We have used it pretty freely for more than three years, and though we find it a remedy of much value, and one for which we have been unable to find a substitute; yet its use has sometimes been attended by effects that should be known to the inexperienced. One objection that has been urged against it is, that the use of a poison so virulent as arsenic, can not be unattended by danger. If properly used, I conceive this objection can never be valid, as the quantity necessary to produce the most desirable result can never be productive of any serious consequences. The power of the remedy should only be a caution to the inexperienced against the use of a quantity, that may be as unnecessary as dangerous. A second objection has been founded on the pain which is said to have resulted from its application to an exposed nerve.

In our first experiments we used simply three parts of arsenic and one of acetate or sulphate of morphine, and contrary to our expectations in nearly all cases where the nerve was much exposed, the application was attended by a considerable amount of pain. When the nervous pulp of a molar tooth was denuded and inflamed, the pain was often excruciating. More recently we have used equal parts of arsenic and morphine. A small quantity of this, say the fifteenth or twentieth of a grain, is sprinkled on a piece of cotton that has previously been saturated with kreosote, and carefully applied to the exposed nerve, after as much of the foreign matter as practical has been cautiously removed from its surface. Over this, white wax is to be applied in a manner to prevent pressure, and the whole permitted to remain five or six hours. If the preparation is applied to more than one tooth at the same time, the patient should be cautious against swallowing his saliva, lest nausea to a greater or less extent be the consequence. Since having used the kreosote, we have found the amount of pain to be very materially diminished, but never entirely wanting if the nervous pulp is much exposed. Where the bony parieties of a tooth is sensitive to such a degree as to make its preparation for a plug painful, this sensibility can always be perfectly removed, and with no pain, by this application. But this practice is not unfrequently followed by results which constitute the chief objection that we desire to point out in this communication. As is recommended by many at the present time, it was our former practice to apply it in all cases where the nervous sensibility of a carious tooth was such as to make its preparation for a plug painful. In teeth that are highly organized, especially in young persons, every dentist knows that a carious surface may be exquisitely sensitive though the nervous pulp may not be exposed. This is especially the case with the incisor and cuspidati. In such cases as we have before said, the application of arsenic will entirely remove the morbid sensibility for the time, and the tooth can be plugged with the greatest facility. But it often occurs that in a few days the whole tooth becomes so exquisitely sensitive, as to be affected by the slightest change of temperature. If the enamel is thin and transparent, the tooth assumes a purple or reddish brown color, and the investing membrane of the fang becomes more or less inflamed as is indicated by pain in occlusion of the jaws. Where the absorption of the poison is slight this appearance subsides in a few days, and the tooth is restored to its original color. In other cases where the morbid effect was more marked, the inflammatory symptoms have continued from one to two months; when the tooth would become perfectly destitute of vitality. The purple color would then be changed to a dark brown, that permanently destroys the beauty of the tooth. In one or two instances I have removed plugs that have been in such a tooth for two years or more, and not the slightest trace of vitality has been discovered. The nerve and blood-vessels through the whole dental canal have been completely destroyed, though they could have been in contact with the preparation only through the medium of absorption. If the enamel of a front tooth is partially transparent, and the patient young, indicating activity of the absorbents, he should bear the pain of having it prepared for a plug without the use of arsenic, rather than endanger its beauty. If the tooth be opaque there is little danger of absorption taking place; but in this case the bony parieties are rarely so sensitive as to render any application necessary. If in the above cases we do not entirely discard the use of arsenic, it would probably be better to continue its application to a particular tooth not more than one hour, and then proceed immediately to cleanse and plug. By this practice, absorption may be to some extent at least prevented, and the desirable results partially, if not wholly, obtained.

If the nervous pulp of an incisor or cuspidatus is exposed, especially in a young person, it is better to destroy it by thrusting a small instrument up the dental canal: as by this means the portion of vitality which is derived from the external periosteum, will, for a time, at least, be retained. If the exposure is slight, the lead or gold cap may be had recourse to. In the bicuspides or molar teeth the arsenious compound may be freely used, provided they have not so far decayed as to render plugging impracticable. In these cases great care should be taken to remove the remains of the disorganized nerve and blood-vessels, and all foreign matter from the dental canal, before the plug is introduced, lest by decomposition of these substances, a gas may be generated, and inflammation of the parts surrounding the tooth, result. If the cavity is of a shape and size to render plugging impracticable, or the lining membrane of the dental canal secretes pus, as sometimes happens, it is better at once to have recourse to extraction.*

* The value of arsenic as an agent for destroying an exposed dental nerve, or the sensibility in a decaying tooth, preparatory to the operation of plugging, has now become well established, but its indiscriminate use, is by no means advisable; the circumstances under which its employment is proper are very correctly set forth in the foregoing communication. The effect which it has, when applied to the teeth of very young persons, as described by Dr. Ide, we have frequently noticed, and for the last two years, have refrained from applying it to the teeth of very young subjects, unless urged to it, by the most pressing necessity. The effect here alluded to, was described by Dr. H. H. Hayden, at the first meeting of the American Society of Dental Surgeons, held in August, 1840, in the city of New York.—*Balt. Ed.*

